# The Biota of Canada: Terrestrial Arthropods

**DOI:** 10.3897/zookeys.819.31621

**Published:** 2019-01-24

**Authors:** David W. Langor, Cory S. Sheffield

**Affiliations:** 1 Natural Resources Canada, Canadian Forest Service, 5320 – 122 St. NW, Edmonton, Alberta, T6H 3S5, Canada Natural Resources Canada, Canadian Forest Service Edmonton Canada; 2 Royal Saskatchewan Museum, 2340 Albert Street, Regina, Saskatchewan, S4P 2V7, Canada Royal Saskatchewan Museum Regina Canada

*This work is dedicated to the visionaries who conceived and created The Biological Survey of Canada*.

Forty years ago, a group of visionary scientists, mostly entomologists and arachnologists, undertook an ambitious project to produce the monograph *Canada and its insect fauna* (Danks 1979). This landmark publication was the first large science product completed by the then-new Biological Survey of Canada (BSC). The large team and ambitious work plan was coordinated, and the product edited, by Hugh V Danks. Hugh’s reputation for scientific rigor, organizational acumen, attention to detail, and synthesis are legendary and inspirational, and were critical to the success of the project. In 573 printed pages, this team of 60 specialists reviewed patterns and determinants of regional diversity, aquatic and terrestrial insect habitats, approaches to documenting the fauna, and the status of each group of terrestrial arthropods in Canada. The rest is history! The monograph has served as a benchmark for biodiversity science in Canada for four decades. The entire work and the individual chapters therein have been cited thousands of times, a testimony to the enormous impact of this product in Canada and beyond. Furthermore, *Canada and its insect fauna* (and many other BSC products to follow over nearly four decades) proves that great things can be achieved by communities of dedicated, capable, and passionate individuals who are willing to push aside sometimes-restrictive institutional impediments, rise above political undercurrents, and focus on delivering together something that is important and lasting. This approach to doing exceptional science continues to define the BSC.

Even after nearly 40 years, the content of *Canada and its insect fauna* is highly valuable. Nonetheless, some of the content of the 39 faunistics chapters, especially the information in the tables, required updating to reflect advances since 1979. Thus, in 2016, the BSC commenced planning a project to update those chapters. However, it was soon realized that the need to re-assess the state of knowledge of species diversity in Canada extended beyond the terrestrial arthropods to the entire biota, as the last assessment was published in 1995 (Mosquin et al. 1995). A number of other organizations were consulted and they endorsed the concept of a new biodiversity assessment for Canada. Thus, the Biota of Canada project was initiated, with the intent of reviewing and assessing the state of biodiversity knowledge of all groups of organisms in Canada in a series of volumes. The terrestrial arthropods were an obvious starting point to develop the ‘proof of concept’ as the 40^th^ anniversary of the BSC was imminent and this was envisioned to be a product to commemorate the occasion.

A Biota of Canada Editorial Committee was established to develop some guidelines for preparation of manuscripts and to ensure that there was a standardised approach in terms of topics covered and data provided. Supported by a set of ‘Instructions to Authors’ and a sample manuscript to distribute to potential authors, the process of soliciting lead authors for papers went remarkably smoothly. Almost all authors first approached agreed to join the writing team, further indicating that the Biota of Canada project was perceived to be of broad value. Most papers were lead by specialists working in Canadian institutions, but for ten organismal groups there were no current or available Canadian experts so expertise was sought and obtained in other countries (China, Czech Republic, Norway, USA). Thus, the Biota of Canada is an international effort.

All authors were asked to review the state of knowledge of the diversity of their taxon in Canada with attention focused on advancements since Canada and its insect fauna was published (1979). Each author was asked to produce a table that provided for each family the following information for the Canadian fauna: number of species reported in 1979, number of described species currently known, number of DNA Barcode Index Numbers (BINs; Ratnasingham and Hebert 2013) attributed to Canadian specimens in the Barcode of Life Datasystems database (BOLD; Ratnasingham and Hebert 2007), the number of additional species expected to eventually be discovered in Canada, the general distribution by ecozone (Federal, Provincial, and Territorial Governments of Canada 2010), and important information sources that have advanced our understanding of the fauna since 1979. Authors were also asked to highlight important gaps and opportunities concerning knowledge of the Canadian fauna. Beyond a few standardized requirements, authors were free to develop their manuscripts as they saw fit. Some provided more detail than others and some provided additional faunal analyses. Together these papers provide a comprehensive overview of the state of knowledge of terrestrial arthropod biodiversity in Canada, and the body of work is analyzed and summarized in an included synthesis paper.

Some groups that were included in Canada and its insect fauna were excluded from the current work, namely tardigrades (water bears), which are not arthropods, and crustaceans. Terrestrial and freshwater crustaceans were covered in two chapters in Danks (1979), Pentastomida and Crustacea, but as the vast majority of crustaceans are marine, this subphylum will be treated in a future volume that includes all other animals in Canada.

It is expected that this work will serve multiple purposes. There is cause to celebrate as our knowledge of the Canadian terrestrial arthropod fauna has advanced significantly over the last 40 years, although not equally so for all groups. There are clearly more decades of focused work required before our fauna is documented well, i.e., on par with the state of knowledge for most European countries, but we expect that this assessment will have immediate value to support our national requirement of reporting on the status of biodiversity in Canada. We hope that the analyses of gaps and needs will help to guide decision-makers who have a mandate to document, report on, and preserve Canadian biodiversity so that future resourcing will be appropriately invested for maximum benefit. Finally, this work represents a marvelous example of the collaborative spirit that is alive and well amongst biodiversity scientists in Canada. We hope that this will inspire others to come along side and contribute to complete the ambitious Biota of Canada publication series.

The strategic direction of this project was guided by the Biota of Canada Editorial Board (Robb Bennett, Jeremy deWaard, José Fernández-Triana, Rémi Hébert, David Langor, Jade Savage, and Cory Sheffield). The editors would like to thank Jeremy deWaard for reviewing or providing data for the DNA barcode portions of almost all papers and the staff of Pensoft, particularly Yordanka Banalieva, Plamen Pankov, and Veselin Kostadinov for their highly competent help in the review and publication process. The entire authorship team thanks the many expert reviewers who provided valuable feedback that improved individual papers and hence the work as a whole. Funding for the publication of this work was provided by the home institutions of authors as well as by the BSC.
